# Characterization of intratissue bacterial communities and isolation of *Escherichia coli* from oral lichen planus lesions

**DOI:** 10.1038/s41598-020-60449-w

**Published:** 2020-02-26

**Authors:** Keumjin Baek, Jaewon Lee, Ahreum Lee, Junho Lee, Hye-Jung Yoon, Hee Kyung Park, Jongsik Chun, Youngnim Choi

**Affiliations:** 1 Department of Immunology and Molecular Microbiology, School of Dentistry and Dental Research Institute, Seoul National University, 101 Daehak-ro, Jongno-gu, Seoul, 03080 Republic of Korea; 2 Department of Oral Pathology, School of Dentistry and Dental Research Institute, Seoul National University, 101 Daehak-ro, Jongno-gu, Seoul, 03080 Republic of Korea; 3 Department of Oral Medicine and Oral Diagnosis, School of Dentistry and Dental Research Institute, Seoul National University, 101 Daehak-ro, Jongno-gu, Seoul, 03080 Republic of Korea; 4 School of Biological Sciences and Institute of Microbiology, Seoul National University, 1 Gwanak-ro, Gwanak-gu, Seoul, 08826 Republic of Korea

**Keywords:** Metagenomics, Infection

## Abstract

Oral lichen planus (OLP) is a chronic T cell-mediated inflammatory disease of unknown etiology. We previously proposed that the intracellular bacteria detected in OLP lesions are important triggering factors for T cell infiltration. This study aimed to identify OLP-associated bacterial species through the characterization of intratissue bacterial communities of OLP lesions. Seven pairs of bacterial communities collected from the mucosal surface and biopsied tissues of OLP lesions were analyzed by high-throughput sequencing of the 16S rRNA gene. The intratissue bacterial communities were characterized by decreased alpha diversity but increased beta diversity compared with those on the mucosal surface. While the relative abundance of most taxa was decreased within the tissues, that of *Escherichia coli* was significantly increased. Four *E. coli* strains were isolated from additional OLP biopsies and verified as K12 strains by whole-genome sequencing. The distribution of *E. coli* in sections of control (n = 12) and OLP (n = 22) tissues was examined by *in situ* hybridization. *E. coli* was detected in most OLP tissues, suggesting its potential role in the pathogenesis of OLP. The oral *E. coli* strains isolated from OLP tissues will be useful to investigate their role as triggering factors for T cell infiltration.

## Introduction

Oral lichen planus (OLP) is a T cell-mediated chronic mucocutaneous disease of unknown etiology and is currently incurable. OLP occurs in 0.5–4% of the global population and appears more prevalent in females than males at a ratio of 1.5:1^1–3^. The main histological features of OLP include band-like lymphocytic infiltration at the superficial part of connective tissue, the presence of liquefaction degeneration in the basal cell layer and the absence of epithelial dysplasia^4^. Although the etiology and pathogenesis of OLP are not completely understood, unknown antigens are thought to trigger T cell infiltration and inflammatory responses of infiltrated lymphocytes such as CD4^+^ and CD8^+^ T cells^5^. What triggers the activation and recruitment of T cells is an important question to find new therapeutics for OLP.

Recently, many researchers have attempted to understand the relationship between human diseases and the human microbiome through high-throughput sequencing technology. To date, four research groups have studied the oral microbiota associated with OLP by sequencing the 16S rRNA gene, and differences in the microbiota of saliva and buccal mucosa between healthy controls and OLP patients have been reported^2,6–9^. We previously reported increased bacterial invasion into the lamina propria, the presence of bacteria within basal epithelial cells and T cells in OLP lesions, and the induction of T cell chemokines by oral bacteria^6^. In addition, Mizuki *et al*. reported that *Mycoplasma salivarium* was detected by immunohistochemistry in the epithelium and lymphocyte infiltrate area in 58.5% of OLP tissues from Japanese patients^10^. These results suggest that oral bacteria may have a role in the pathogenesis of OLP. However, clear evidence for the causality of bacterial infection and a link to a specific species are lacking. The aim of this study was to identify OLP-associated bacterial species through the characterization of bacterial communities within the tissues of OLP lesions.

## Results

### Decreased alpha diversity and enrichment of *Escherichia coli* within the tissues compared with the mucosal surface

**Figure 1:**
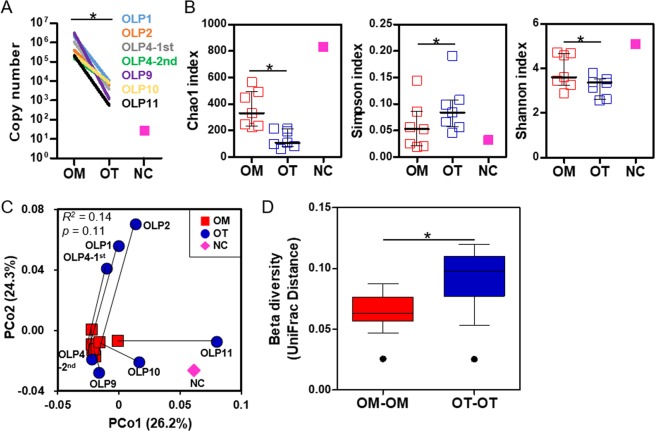
Alpha and beta diversities of the mucosal surface and intratissue communities within OLP lesions. Bacterial communities from the mucosal surface (OM) and biopsied tissues (OT) of OLP lesions were analyzed by sequencing the bacterial 16S rRNA gene. For the negative control (NC), DNA was extracted without sample using a DNA extraction kit and subjected to sequencing of the bacterial 16S rRNA gene. (**A**) Total bacterial load in each sample was estimated by real-time PCR using universal primers targeting the bacterial 16S rRNA gene. (**B**) The species richness, evenness, and diversity of the two communities were estimated by the Chao1, Simpson and Shannon indexes, respectively. The horizontal lines and error bars represent medians and interquartile ranges, respectively. (**C**) A PCoA plot was generated using the weighted UniFrac metric with normalization for read counts. *p* value by PERMANOVA analysis. (**D**) The intersubject UniFrac distances of the OM and OT communities were obtained using a weighted metric and presented as box and whisker plots. *p < 0.05 by two-tailed Wilcoxon signed-rank test.

To identify OLP-associated bacterial species, bacterial communities present inside the oral tissue (OT: n = 9) and those colonized on the oral mucosal surface (OM: n = 7) of OLP lesions were analyzed by high-throughput sequencing of the 16S rRNA gene. Detailed clinical information and the sequencing platform are presented in Table 1. Because two different sequencing platforms were used for the current study, only paired analyses using the Wilcoxon signed-rank test were performed for seven OM-OT pairs. When total bacterial loads were estimated by real-time PCR, copy numbers present in the OT samples were decreased by 2 to 3 orders of magnitude compared to the OM samples. In the negative control sample, approximately 75 copies were detected in the total elution volume (Fig. 1A). A total of 479,058 filtered reads with an average length of 408 bp were obtained from the 14 samples, which presented ≥98.4% coverage for each sample. The species richness, evenness, and diversity determined by the Chao1, Simpson, and Shannon indexes were significantly decreased in the OT communities compared to the OM communities. Meanwhile, the negative control sample presented very high species richness and diversity (Fig. 1B). In the UniFrac-based principal coordinates analysis (PCoA) plot, the OM communities clustered to each other, but the OT communities were dispersed, partially overlapping with the OM communities (Fig. 1C and Supplementary Fig. S1). As a result, the OT group had a significantly increased UniFrac distance compared to the OM group (Fig. 1D). The permutational multivariate analysis of variance (PERMANOVA) test revealed that the OT communities were not significantly different from the OM communities (*r*^2^ = 0.14, *p* = 0.11).

**Table 1:** The demographic and clinical information of subjects.

No.	Age	Sex	REU^a^ scoring		Sequencing		*E. coli* isolation
			REU	Score	Mucosa	Tissue	
OLP1	67	F	R5E3	9.5	G^b^	G	ND^d^
OLP2	68	F	R2E2	5	G	G	ND
OLP4-1st	55	F	R2E4	8	G	G	ND
OLP4-2nd	57	F	R5E2	8	M^c^	M	ND
OLP5	74	M	R2E1	3.5	ND	ND	K12-5.1
OLP6	61	F	R7E5	14.5	ND	G	ND
OLP7	60	F	R1E3U1	7.5	ND	ND	K12-7.1
							K12-7.2
							K12-7.3
OLP8	52	F	R2E5	9.5	ND	G	ND
OLP9	76	M	R3E4U1	11	M	M	ND
OLP10	54	M	R6E6U1	17	M	M	ND
OLP11	59	M	R5E5	12.5	M	M	ND

^a^R, reticulation; E, erythema; U, ulceration, ^b^G, Roche 454 GS FLX Titanium System; ^c^M, Illumina MiSeq System; ^d^ND, not done.

**Figure 2:**
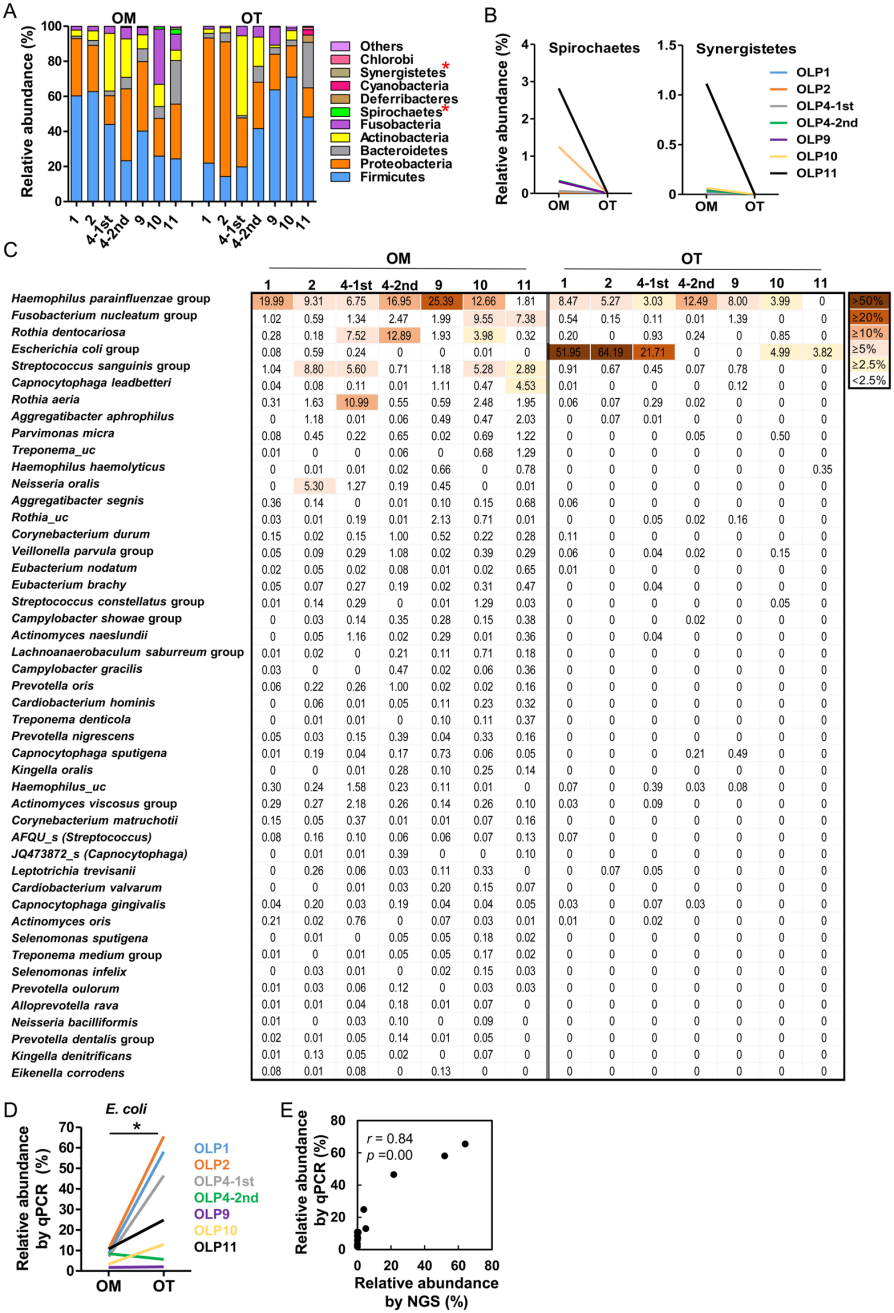
Differences in relative abundance between the mucosal surface and intratissue communities at the phylum and species levels. (**A**) The members of the top 10 phyla are shown. (**B**) Two phyla were differentially distributed between the two communities. (**C**) The relative abundances of 47 species/phylotypes differently distributed (*p* < 0.05 by two-tailed Wilcoxon signed-rank test) between two groups are shown as a heat map. (**D**) The relative abundance of *E. coli* was estimated by real-time PCR using universal- and *E. coli*-specific primers. (**E**) Correlation plot of relative abundances obtained by high-throughput sequencing and qPCR methods. *p < 0.05 by two-tailed Wilcoxon signed-rank test. *r* and *p* values by Spea*r*man’s rank correlation test.

To determine differences in the composition of bacterial communities, only the taxa with relative abundance ≥0.1% in at least one sample were analyzed. At the phylum level, differences in the bacterial compositions of the OM and OT communities obtained from each patient were evident (Fig. 2A). However, only decreases in Spirochaetes and Synergistetes in the OT group compared to the OM group were consistently observed (Fig. 2B). At the genus level, the relative abundances of *Escherichia, Acinetobacter*, and *Sphingomonas* were significantly increased, but those of 19 genera were significantly decreased in the OT group compared with the OM group (Table 2). At the species level, 47 species were differentially distributed between the two groups. Among them, only the *E. coli* group was enriched in OT communities (Fig. 2C). Interestingly, the *E. coli* group was not detected in the OLP 4-2nd tissue, although it comprised 21.7% in the OLP4-1st tissue. To confirm the sequencing data, the relative abundance of the *E. coli* group was quantitated by real-time PCR, which showed a strong positive correlation with the result obtained by sequencing (*r* = 0.84, Fig. 2D,E). Among the 822 operating taxonomic units (OTUs) identified in the negative control sample, 735 OTUs were not detected in any OLP samples. When we defined contaminants as the taxa that were detected in only one OLP sample together with the negative control, only the OLP11 OT sample had contaminants among its dominant (>2.5% abundance) species (Supplementary Fig. S2). *M. salivarium*, the species detected in 58.5% of OLP tissues from Japanese patients^10^, was found in three OM samples at <0.1% but in none of the OT samples.

**Table 2:** Relative abundance (%)^a^ of genera differently distributed between the mucosal surface and intratissue communities.

Genus	OM (*n* = 7)	OT (*n* = 7)	*p* value^b^
*Haemophilus*	15.21 (4.16–27.26)	5.27 (0.35–14.18)	0.018
*Neisseria*	7.29 (2.31–18.08)	3.64 (0.09–11.95)	0.043
*Fusobacterium*	2.12 (1.45–9.68)	0.52 (0–1.52)	0.018
*Escherichia*	0.01 (0–0.61)	4.99 (0–65.23)	0.043
*Corynebacterium*	1.15 (0.07–3.62)	0 (0–0.16)	0.018
*Treponema*	0.15 (0.01–2.80)	0 (0–0)	0.018
*Aggregatibacter*	0.59 (0.26–2.73)	0 (0–0.07)	0.018
*Actinomyces*	1.41 (0.88–5.48)	0.37 (0–1.70)	0.043
*Parvimonas*	0.5 (0.02–1.22)	0 (0–0.5)	0.018
*Fretibacterium*	0.04 (0–1.11)	0 (0–0)	0.028
*Eubacterium_g11*	0.19 (0.02–0.47)	0 (0–0.04)	0.018
*Cardiobacterium*	0.08 (0–0.41)	0 (0–0)	0.028
*Acinetobacter*	0 (0–0.04)	0.06 (0–1.0)	0.028
*Saccharimonas*	0.1 (0.03–0.26)	0 (0–0.05)	0.028
*Kingella*	0.14 (0.03–0.33)	0 (0–0.22)	0.043
*Sphingomonas*	0 (0–0)	0.13 (0–1.10)	0.043
*Johnsonella*	0.02 (0–0.27)	0 (0–0)	0.028
*Catonella*	0.03 (0–0.15)	0 (0–0)	0.018
*Mycoplasma_g4*	0.01 (0–0.20)	0 (0–0)	0.043
*Dialister*	0.02 (0–0.15)	0 (0–0)	0.028
*Eikenella*	0.02 (0.01–0.13)	0 (0–0)	0.018
*Atopobium*	0.02 (0–0.12)	0 (0–0.07)	0.043

^a^Relative abundance expressed as median (minimum – maximum).

^b^By two-tailed Wilcoxon signed-rank test.

### Isolation of *E. coli* strains from OLP tissues

**Figure 3:**
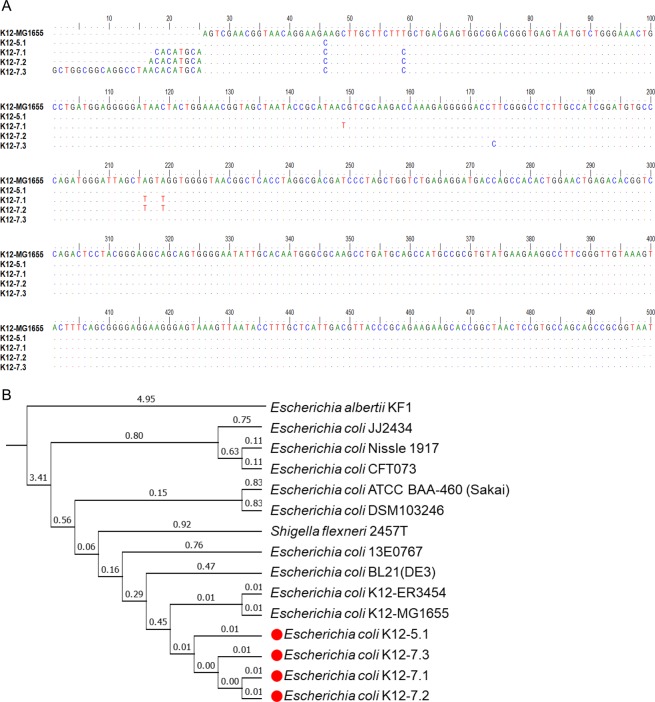
Isolation and identification of clinical isolates from tissues of OLP lesions. (**A**) Aligned nucleotide sequences of the 16S rRNA gene from four clinical isolates and *E. coli* K-12 MG1655. (**B**) Phylogenic analysis of 15 strains in the *E. coli* group. The numbers on the branches show the branch length.

We previously reported that a key periodontal pathogen, *Porphyromonas gingivalis*, invades gingival tissues and is highly enriched in the intratissue bacterial communities at periodontal lesions^11,12^. Similarly, a species enriched within the tissues of OLP lesions may have a role in the pathogenesis of OLP. However, the 16S rRNA gene sequence of the *E. coli* group did not distinguish *E. coli*, *E. albertii*, *E. fergusonii*, *and four Shigella species*. Furthermore, the genetic diversity of *E. coli* is enormous^13^. To investigate the potential association of *E. coli* with OLP, *E. coli* was isolated from the biopsies of two OLP patients: one strain from patient OLP5 and three strains from patient OLP7. By sequencing the 16S rRNA gene (Fig. 3A) and subsequent whole genome sequencing, the isolated strains were verified as *E. coli* K12 strains and named K12-5.1, K12-7.1, K12-7.2, and K12-7.3. The oral *E. coli* strains were very close to each other and relatively close to K12-MG 1655, a type strain isolated from human feces, or to the laboratory strain BL21. However, all strains were genetically unique (Fig. 3B). We also tried many times to isolate *E. coli* from the buccal swabs of healthy individuals, but all attempts failed.

### Invasion of human oral keratinocytes by oral *E. coli* strains

**Figure 4:**
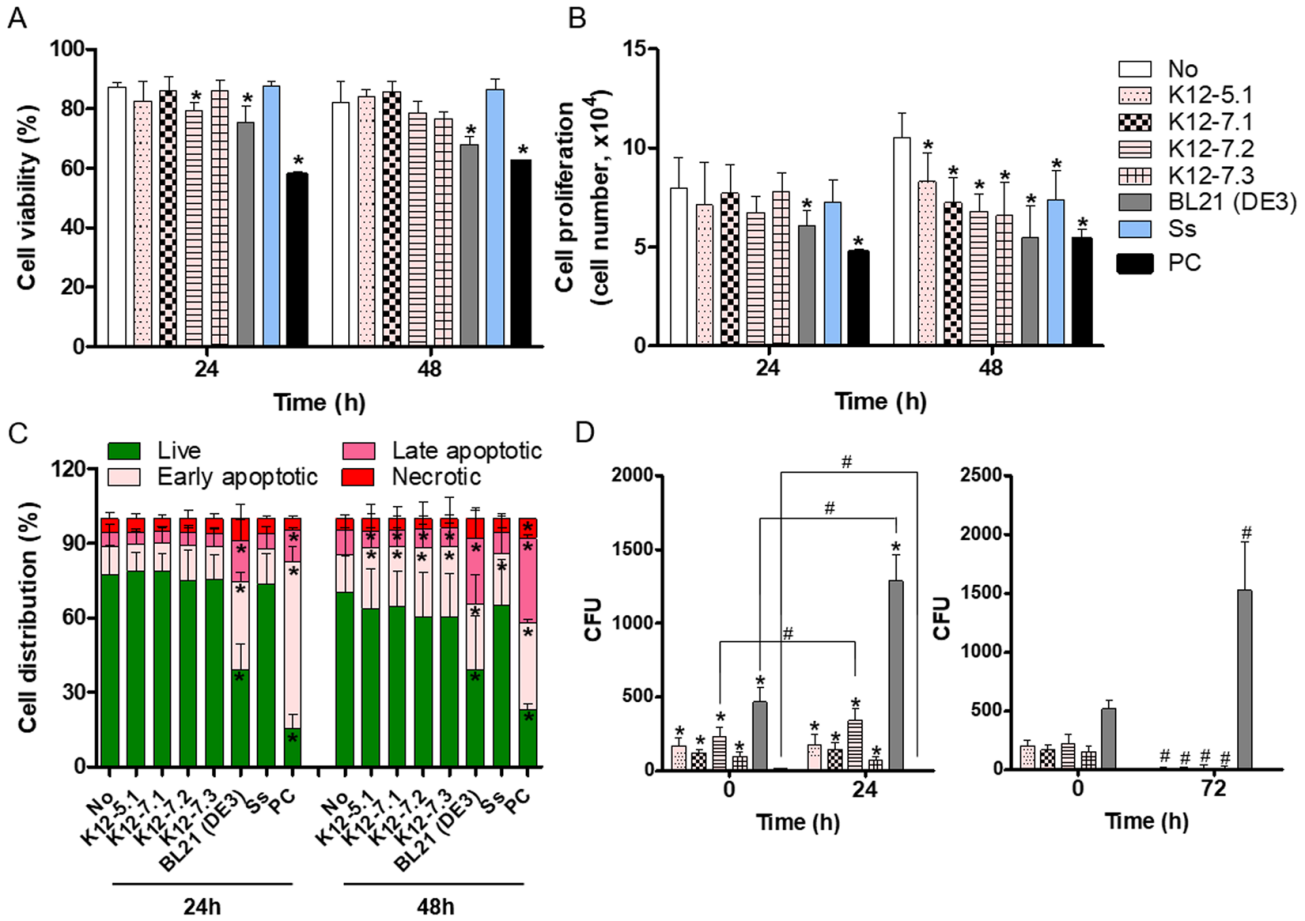
Invasion of HOK-16B cells and induction of apoptosis by oral *E. coli* strains. HOK-16B cells were infected with oral *E. coli* strains and *S. salivarius* (Ss) at an MOI of 1000. As a positive control (PC), cells were treated with 0.5 µg/ml staurosporine. (**A**,**B**) Cell viability and proliferation were measured by trypan blue enumeration. *p < 0.05 compared to untreated cells by t-test. (**C**) The apoptotic stage was measured by flow cytometry using Annexin V-PI staining. *p < 0.05 compared to untreated cells by t-test. (**D**) HOK-16B cells were infected with bacteria at an MOI of 1000 for 2 hours, and the infected cells were treated with gentamicin for 1 hour. The cells were then lysed immediately or further incubated for 24 hours in antibiotic-free medium. The cell lysates were cultured on LB agar plates. *p < 0.05 compared to Ss by Mann-Whitney U test, ^#^*p* < 0.05 by 2-tailed Wilcoxon signed-rank test. All graphs present the mean and standard deviation of two experiments performed in triplicate.

To study the potential role of oral *E. coli* strains in the pathogenesis of OLP, the effects of *E. coli* on the viability and proliferation of immortalized human oral keratinocyte (HOK-16B) cells were examined by trypan blue enumeration. Oral *E. coli* strains did not affect the viability of HOK-16B cells greatly (Fig. 4A) but significantly decreased the proliferation of host cells at 48 hours after infection (Fig. 4B). However, this inhibition of proliferation was also observed by *Streptococcus salivarius*, one of the most abundant commensals on oral mucosa. Staining with Annexin-V and propidium iodide (PI) further verified that infection with oral bacteria induced increases in the percentage of early apoptotic cells defined by Annexin V^+^PI^−^ at 48 hours after infection (Fig. 4C). Interestingly, *E. coli* BL21, a laboratory strain used for recombinant protein production, presented cytotoxicity from 24 hours after infection that coincided with the increases in both early and late apoptotic cells (Fig. 4A–C).

We also evaluated the ability of oral bacteria to invade and survive within HOK-16B cells by an antibiotic protection assay. All oral *E. coli* strains could invade HOK-16B cells and survive within the cells for 24 hours, but less than 8% of the internalized *E. coli* survived at 72 hours after infection. *E. coli* BL21 also efficiently invaded HOK-16B cells and survived even at 72 hours after infection. In contrast to *E. coli*, *S. salivarius* hardly invaded HOK-16B cells and did not survive within the host cells (Fig. 4D).

### Detection of *E. coli* in control and OLP tissues by *in situ* hybridization

**Figure 5:**
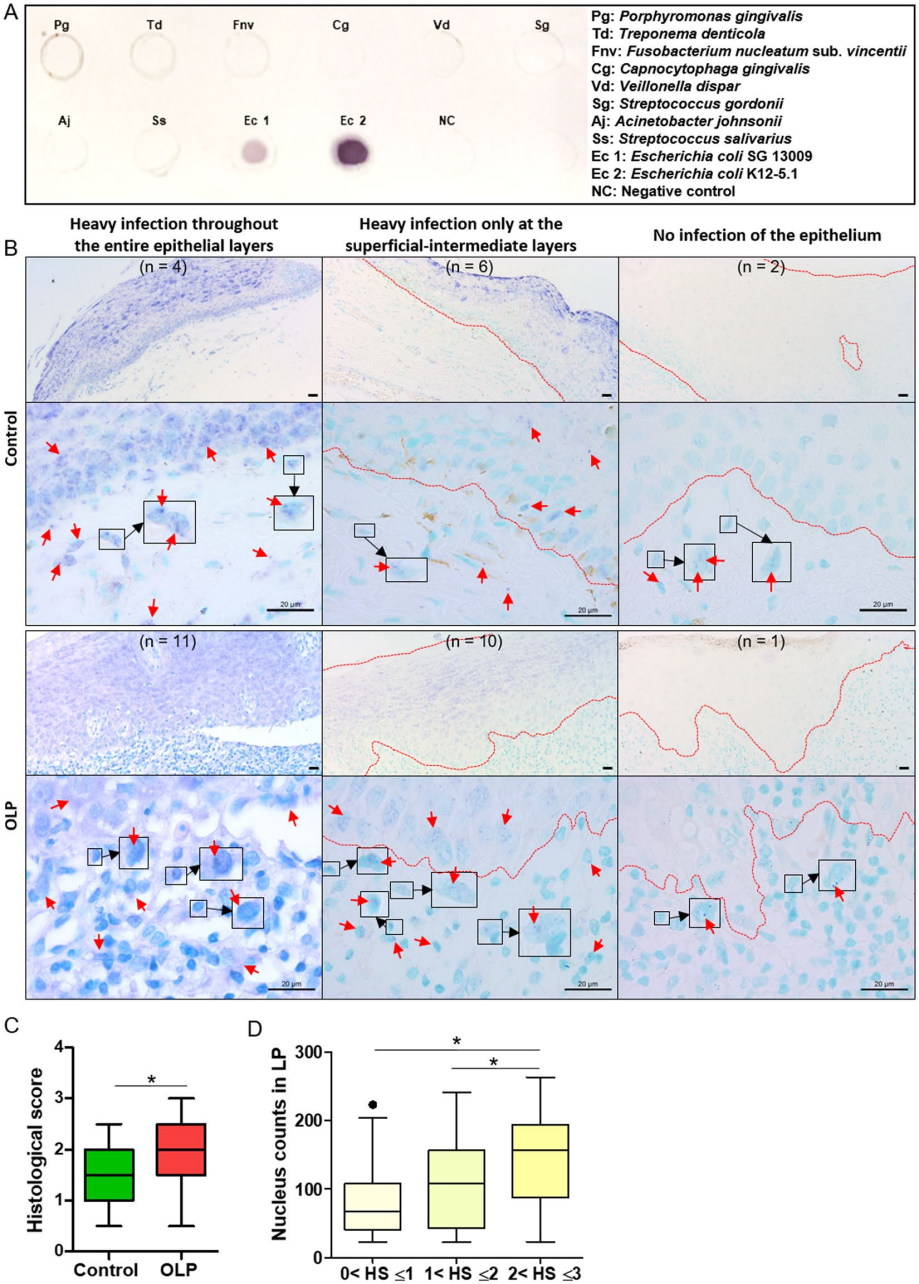
Detection of *E. coli* in control and OLP tissues by *in situ* hybridization (**A**) The specificity of the digoxigenin-labeled probe for *E. coli* was confirmed by dot blot assay. Bacterial gDNA from nine bacterial species was used for the specificity test. (**B**) Representative *in situ* detection patterns of *E. coli* in control (n = 12) and OLP (n = 22) tissues. The numbers in parentheses indicate the number of samples with a similar pattern in the epithelium. In the OLP tissue presented in the left panel, all lamina propria cells had bacterial signals. Representative *E. coli*-infected lamina propria cells are enlarged in squares. Scale bars, 20 µm; red arrows, *in situ* signals for *E. coli*; dotted red lines, the outline of the epithelium. (**C**) The frequency of lamina propria cells infected with *E. coli* was scored (score 0, signal absent; score 1, <30% cells harboring bacterial signal; score 2, 30–60% cells harboring bacterial signal; score 3, >60% cells harboring bacterial signal). *p < 0.05 by Mann-Whitney U test. (**D**) The number of cells in the lamina propria are plotted with the histological score (HS). *p < 0.05 by Kruskal-Wallis test, followed by Mann-Whitney U test.

The presence of *E. coli* within control (n = 12) and OLP (n = 22) tissues was examined by *in situ* hybridization using an *E. coli*-specific probe (Fig. 5A). Various levels of *E. coli* were detected in the epithelium and lamina propria of both control and OLP tissues. *E. coli* distribution within the epithelium could be divided into three patterns: strong signals throughout the entire epithelial layers, strong signals at the superficial-intermediate layers but weak signals at the basal layer, and no signals (Fig. 5B left, middle, and right, respectively). There was no significant difference in the patterns of *E. coli* distribution within the epithelium between the control and OLP groups. In the lamina propria, *E. coli* was detected in all control and OLP samples, although very few signals were detected in several samples (Fig. 5B right). Although the bacterial signal intensities of the areas that included both epithelia and lamina propria were not different between the groups (Supplementary Fig. S3), the degree of lamina propria cells harboring bacterial signals was increased in OLP tissues compared with control tissues (Fig. 5C). In addition, a histologic score >2 was associated with an increased number of cells in the lamina propria (Fig. 5D).

## Discussion

In the present study, we characterized the intratissue bacterial communities of OLP lesions and reported the enrichment and isolation of oral *E. coli* strains from OLP tissues.

The intratissue bacterial communities of OLP lesions were characterized by decreased alpha diversity but increased beta diversity compared with the communities on the mucosal surface. While the relative abundance of most taxa was decreased within the tissues, the relative abundance of *E. coli* was significantly increased, comprising 3.8% to 64.2% in five out of seven communities. *Haemophilus parainfluenzae* was present at 3% to 12.5% in six out of seven tissue samples, although its relative abundance was significantly decreased compared to that on the surface. *E. coli* and *H. parainfluenzae* were also dominant species in the intratissue community of OLP8 that was excluded from the statistical analysis (Supplementary Fig. S2). In addition to *E. coli* and *H. parainfluenzae*, three to 15 species were identified as dominant species in each tissue sample, but those species often did not overlap among samples, explaining the increased beta diversity. Even the two tissue samples obtained from OLP4 in the 2-year term had different dominant species.

When the mucosal communities of OLP lesions were compared with those of healthy individuals from a previous study^6^, decreased relative abundances of the phylum Firmicutes, genus *Escherichia*, and several streptococcal species but increases in the relative abundances of several gingivitis/periodontitis-associated and *Leptotrichia* species in OLP were repeatedly observed (Supplementary Fig. S4). Among the 23 species/phylotypes that were enriched in the OLP lesions, *H. parainfluenzae*, KV831974_s (*Rothia*), *Streptococcus sinensis*, *H. parahaemolyticus*, *Porphyromonas pasteri*, and PAC001356_s (*Leptotrichia*) were found to be dominant species (Supplementary Fig. S2). Interestingly, the relative abundance of *E. coli* on the mucosal surface of OLP lesions was significantly decreased compared to that of healthy individuals both in the current and previous studies; thus, the enrichment of *E. coli* within OLP tissues was a unique phenomenon.

The enrichment of *E. coli* within OLP tissues was completely unexpected. *E. coli* are usually known as gut commensals of humans and animals but also include diverse pathogens that cause diseases in intestinal or urinary tracts^14^. The presence of *E. coli* in the buccal mucosa, supragingival plaque, and tongue dorsum of healthy individuals has been reported in several oral microbiome studies^6,15,16^. As *E. coli* was detected in our negative control sample at an abundance of 0.01% (Supplementary Fig. S2), *E. coli* DNA is a common contaminant of many different DNA isolation kits^17^. However, isolation of genetically unique oral *E. coli* strains from OLP tissues strongly suggests that the *E. coli* enriched within OLP tissues is not a contaminant. Importantly, bacterial contaminants were detected only in the OLP11 tissue sample, which had the lowest bacterial load (574 copies). During the PCR and sequencing of DNA samples, the endogenous and contaminated bacterial DNA copies would compete each other, and the contaminated bacterial DNA would be preferentially detected when the sample contains a low endogenous bacterial load.

The oral *E. coli* strains presented a similar degree of cytotoxicity on oral keratinocytes in culture with the oral commensal *S. salivarius* but less cytotoxicity than the *E. coli* BL21 strain. Importantly, *E. coli*, but not *S. salivarius*, invaded oral keratinocytes and survived at least 24 hours. Type I pili of uropathogenic *E. coli* mediate invasion into host cells through interaction with β1 and α3 integrin receptors^18^. Not only the uropathogenic strain (CFT703) but also all oral and BL21 strains contained genes required for the synthesis of type I pili, including the FimH adhesin (Supplementary Fig. S5). Human oral keratinocytes normally express β1 and α3 integrins^19^, supporting invasion by *E. coli*. Even in nonphagocytic cells, except for some pathogens, internalized bacteria normally enter an endocytic pathway and ultimately undergo endolysosomal degradation^20,21^. Whereas the number of intracellular *E. coli* BL21 strain increased at 72 hours compared to 24 hours after infection, only a small proportion of internalized oral *E. coli* strains survived within the oral keratinocytes 72 hours after infection, suggesting relatively low virulence of the oral strains. Considering the constant shedding of cells from the oral epithelium, the oral *E. coli* strains are unlikely to reach the basal cell layer of the oral epithelium *in vivo*, unless epithelial barriers are severely disrupted, the normal physiology of the endocytic pathway is damaged, or the virulence of *E. coli* is changed. Nonetheless, *E. coli* was observed at the basal cell layer in 10 out of 12 control and 21 out of 22 OLP tissues. In particular, four control and 11 OLP tissues presented strong *E. coli* signals throughout the entire layers of epithelium. Notably, the clinical reasons for performing biopsy of the four control tissues were suspected OLP, hyperkeratosis, a nodular mass, and black pigmentation. Whether such clinical conditions contribute to epithelial infection with *E. coli* is not clear. However, it is evident that *E. coli* can often reach the basal layer of the oral epithelium not only in OLP tissues but also in those with a histologically normal appearance. Since the diagnosis of control tissues was based on H&E staining alone, there is the possibility that they had unrecognized abnormalities.

It is mysterious that no inflammatory infiltration was observed in the four control tissues despite the strong infection of the epithelium with *E. coli*. This may be attributed to the fact that oral keratinocytes produce much less chemokines than monocytes or CD4 T cells in response to bacterial challenge *in vitro*^6^. This was supported by our observation that there was a significant increase in the number of infiltrated cells when >60% lamina propria cells were infected with *E. coli*. This finding also coincides with the previous report that the levels of bacteria detected within the lamina propria had a positive correlation with the levels of infiltrated T cells^6^. Therefore, infection of lamina propria cells seems to be important for recruiting T cells. Interestingly, one control tissue from a patient with suspected OLP presented that almost all lamina propria cells were infected with *E. coli*, and a small area of lymphocytic infiltration was observed (Supplementary Fig. S6). According to medical records, the patient received treatment with dexamethasone gargle and nystatin for two years. It is tempting to speculate that this control tissue may present the initial stage of OLP.

The characteristics of the intratissue bacterial communities in OLP lesions were quite different from those in periodontitis lesions^11^. The bacterial communities within the gingival tissues had similar alpha diversities but decreased beta diversities compared with the plaque communities. In addition, *Fusobacterium nucleatum* and *P. gingivalis*, the two species highly enriched within gingival tissues, accumulate in the subgingival plaque when the periodontal status converts from health to periodontitis^11,22^. Although periodontitis is a polymicrobial infection, *P. gingivalis* serves as a multipotent pathogen that drives the dysbiosis of the subgingival microbiome, damages the epithelial barriers with powerful proteinases, invades gingival tissues, and evades host immunity to cause persistent infection^13,23–26^. Whether *E. coli* can play multiple roles in OLP is an interesting question to be answered. If bacteria have a role in the pathogenesis of OLP, it is likely to be polymicrobial like most oral diseases. The increased beta diversity of intratissue communities suggests that different species can be involved from patient to patient. In addition, the possibility that the dysbiosis of mucosal oral microbiota and bacterial invasion of tissues observed in OLP is the result of epithelial barrier disruption caused by other reasons cannot be excluded.

One of the limitations of this study is the small sample size used for the microbiota analysis. Although we identified differences between mucosal and intratissue communities through parallel comparison, more samples from a new cohort need to be studied in the future. There is a limitation in comparing microbiota data obtained by two different sequencing platforms. We minimized this limitation by comparing OM-OT pairs obtained by the same sequencing platform.

In conclusion, bacterial communities present within the tissues of OLP lesions are less complex and have a higher interpatient variability than the communities colonized on the mucosal surface. *E. coli*, which is enriched within tissues, invades oral keratinocytes and is observed in most OLP tissues, may play an important role in the pathogenesis of OLP, although other species may also be involved. The oral *E. coli* strains isolated from OLP tissues will be useful to investigate their role as triggering factors for T cell infiltration.

## Methods

### Human samples

This study was performed in accordance with the Helsinki Declaration under procedures approved by the Institutional Review Board at the Seoul National University Dental Hospital (IRB No. CRI 15023 and CRI17007) and at Seoul National University, School of Dentistry (S-D20180026). Informed consent was obtained from all 10 patients with OLP. All enrolled patients had no history of antibiotics or steroid treatment within one month. Patients with a low unstimulated whole salivary flow rate (≤0.1 ml/minute) and smokers were excluded. A semiquantitative REU (reticulation/erythema/ulceration) scoring system was adapted to examine the severity of the lesion.

Reticular lesions with or without erythema but with no ulceration located at the buccal mucosa were chosen. First, bacteria from the mucosal surface of the lesion were sampled by placing a sterilized 20 mm × 20 mm polyvinylidene difluoride membrane (Merck Millipore, Burlington, MA, USA) on the buccal mucosa for 30 seconds. Then, two punch biopsies of 4 mm were obtained: one was used to make a tissue block for pathologic diagnosis, and the other was used to extract DNA for microbiota analysis. Mucosal bacteria and biopsies were obtained two times from patient OLP4 over 2 years to rule out tumorigenesis. In the case of OLP6 and OLP8, sampling of the mucosal surface was omitted by mistake. Among the 11 biopsy samples from the OLP patients, two samples (OLP5 and OLP7) were not included for sequencing but were used to isolate *E. coli*.

For *in situ* hybridization, sections of 12 control and 22 OLP tissues, which included tissues collected from the patients recruited for the microbiota study, were obtained. The control samples were biopsies performed for various reasons, but no diagnostic abnormalities were recognized based on the examination of H&E stained sections.

### Preparation of bacterial DNA samples

Bacterial genomic DNA was isolated from buccal mucosal swabs or tissues using a commercial kit for soil bacteria (MOBIO Laboratories, Carlsbad, CA, USA). The tissues were pretreated before DNA extraction as previously described^11^. Briefly, the tissues were washed and incubated with 1 ml of PBS containing lysozyme (300 µg/ml) and antibiotics (penicillin, streptomycin, and gentamicin) at 37 °C for 1 hour. After washing, the tissues were then incubated with DNase I to digest bacterial DNA on the surface of the tissues. After heat inactivation of DNase I, the tissues were homogenized and subjected to bacterial DNA extraction. In addition to these bacterial DNA samples, a negative control in which DNA extraction had been performed without sample was included.

### Analysis of bacterial communities

Genomic DNA of the buccal mucosa and tissue samples was subjected to bacterial community analysis. As listed in Table 1, the V1-V3 or V3-V4 hypervariable regions of the bacterial 16S rRNA gene were amplified by PCR, and then the PCR products were sequenced using a 454 GS FLX Titanium (Roche Applied Science, Bavaria, Germany) or an Illumina MiSeq (Illumina, San Diego, CA, USA) sequencing system at ChunLab Inc. (Seoul, Korea), respectively. We had to change the sequencing system because reagents for the 454 GS FLX Titanium were discontinued during the study. The negative control sample was sequenced using Illumina MiSeq. Processing and analysis of the sequences were performed using CLcommunity™ software provided by ChunLab Inc. The bioinformatics used in the CLcommunity™ software was previously reported^12^. The sequence data are available in the NCBI Sequence Read Archive under the accession number SRP175120.

### Quantification of total bacterial loads and *E. coli*

Real-time PCR was performed in a 20 μl reaction mix containing 2 μl of bacterial genomic DNA, SYBR *Premix Ex Taq*, ROX Reference Dye II (Takara Bio, Otsu, Japan), and each primer. Universal (forward, 5′-AGT CAC TGA CGA GTT TGA TCM TGG CTC AG-3′; reverse, 5′-CAG TGA CTA CWT TAC CGC GGC TGC TGG-3′) and *E. coli*-specific primers (forward, 5′- CCA TGC CGC GTG TAT GAA GA-3′; reverse, 5′- AGA TGC AGT TCC CAG GTT GAG-3′) targeting the bacterial 16S rRNA gene were used. *E. coli* K-12 5.1 genomic DNA was used to generate standard curves. The copy numbers of the 16S rRNA genes of total bacteria and *E. coli* were estimated using standard curves, and the relative abundance of *E. coli* was calculated. Real-time PCR using universal primers and reagent control always resulted in a positive Ct value of approximately 32, suggesting contamination inherent to PCR. For quantitation of total bacterial loads, the Ct value of all DNA samples, including those for standard curves, was corrected for the Ct value of the reagent control.

### Isolation of *E. coli* from OLP lesions

To isolate the clinical strains of *E. coli* located inside the tissues of OLP lesions, biopsies from two patients were treated with lysozyme and antibiotics as mentioned above. After washing, the tissues were homogenized and suspended in 5 mL of tryptic soy broth (TSB). Samples in TSB were incubated aerobically at 41.5 °C for 24 hours for pre-enrichment. Following incubation, the enrichments were streaked on eosin methylene blue agar and incubated at 37 °C overnight. Colonies on agar plates were inoculated into TSB and cultured at 37 °C overnight. Four clinical isolates were confirmed as *E. coli* by sequencing the 16S rRNA gene followed by whole genome sequencing using Illumina MiSeq at ChunLab Inc. The assembled genomes of the isolated strains were compared with those of other *E. coli* strains using Comparative Genomics (CG) software provided at the integrated database EzBioCloud^27^.

### Epithelial cell viability, proliferation, and apoptosis

HOK-16B cells (RRID: CVCL_B405) were maintained in keratinocyte growth medium (KGM) containing supplementary growth factors (Lonza, Basel, Switzerland) at 37 °C in a water-saturated atmosphere of 5% CO_2_. To examine the effect of the clinical isolates on the cell viability and proliferation of HOK-16B cells, cells (6 × 10^4^ cells) were infected with four clinical isolates of *E. coli*, *E. coli* BL21 (Thermo Fisher Scientific, Waltham, MA, USA), and *S. salivarius* KCTC 5512 (Korean Collection for Type Culture, Jeongeup, Jeollabuk-do, Korea) at a multiplicity of infection (MOI) of 1000 in KGM without antibiotics for 2 hours. After 2 hours, 50 μg/ml and 100 μg/ml gentamicin were added to kill extracellular *E. coli* and *S. salivarius*, respectively. After incubation for the indicated times, the cells were detached by trypsin-EDTA and enumerated after trypan blue staining. To analyze apoptosis of cells, the detached cells were also washed with Annexin-binding buffer (10 mM HEPES, 140 mM NaCl, and 2.5 mM CaCl_2_, pH 7.4) and stained with 5 μl of Annexin V-Alexa Fluor™ 488 conjugate (Life Technologies, Carlsbad, CA, USA) and 5 μg/ml PI in 100 μl of Annexin-binding buffer for 15 min at room temperature. After adding 400 μl of Annexin-binding buffer, the stained cells were analyzed by FACSCalibur™ (BD Biosciences, San Diego, CA, USA).

### Antibiotic protection assay

To examine the ability of bacteria to invade oral epithelial cells, HOK-16B cells plated in 24-well plates were infected with clinical isolates of *E. coli* and *S. salivarius* KCTC 5512 at an MOI of 1000 for 2 hours in the absence of antibiotics. After 2 hours, the cells were treated with gentamicin for 1 hour to kill the extracellular bacteria. Then, the infected cells were subjected to lysis with sterile distilled water containing 0.5% saponin immediately or after further culture in new antibiotic-free culture medium for 24 or 72 hours. The lysates were plated on LB agar plates and cultured overnight. The number of bacteria that survived within the cells was expressed as colony forming units (CFUs).

### *In situ* hybridization

A 244-bp DNA fragment chosen based on the well conserved area located between V3 and V4 of *E. coli* 16S rRNA was amplified by PCR using the *E. coli*-specific primers and gDNA extracted from the isolated oral *E. coli* strains. The amplified products were labeled with digoxigenin (DIG)-11-dUTP by random priming using a commercial DIG DNA Labeling and Detection Kit (Roche Applied Science, Bavaria, Germany). After the specificity and sensitivity of the DIG-labeled probe were confirmed, *in situ* hybridization was performed using 4 μm paraffin-embedded sections as previously described^11^. As a negative control, hybridization was performed with the labeled probe mixed with a 10-fold excess amount of unlabeled probe. Four areas in the lamina propria per sample were photographed at 400x magnification, and the frequency of cells with bacterial signals was blindly scored from 0 to 3 by two students (score 0, signal absent; score 1, <30% cells harboring bacterial signal; score 2, 30–60% cells harboring bacterial signal; score 3, >60% cells harboring bacterial signal). In addition, the number of nuclei in the same image was counted.

### Statistical analysis

The Wilcoxon signed-rank test was used to compare various parameters between the mucosal surface and intratissue bacterial communities. The t-test was used to analyze the data from cell viability, proliferation, and apoptosis assays using HOK-16B cells. The Wilcoxon signed-rank test, Kruskal Wallis test and Mann-Whitney U-test were used to analyze the results of the antibiotic protection assay and *in situ* hybridization. Spearman’s rank correlation test was used to determine the correlation of relative abundance between 2 analysis methods. All statistical analyses were performed using SPSS Statistics 25 software (SPSS Inc., Chicago, IL, USA). In addition, PERMANOVA was performed with the adonis function from the vegan package in R. Significance was set at *p* < 0.05.

## Supplementary information

Supplementary Information.
