# Supplementary material for: Characterization of intratissue bacterial communities and isolation of Escherichia coli from oral lichen planus lesions
Source: Sci Rep. 2020 Feb 26;10:3495. doi: 10.1038/s41598-020-60449-w (PMC7044275; doi:10.1038/s41598-020-60449-w)
Supplement: Supplementary file 1 — Supplementary Information. [file 41598_2020_60449_MOESM1_ESM.pdf]

Characterization of intratissue bacterial communities and isolation of *Escherichia coli* from oral lichen planus lesions

Keumjin Baek[1], Jaewon Lee[1], Ahreum Lee[1], Junho Lee[1], Hye-Jung Yoon[2], Hee Kyung Park[3], Jongsik Chun[4*], Youngnim Choi[1*]

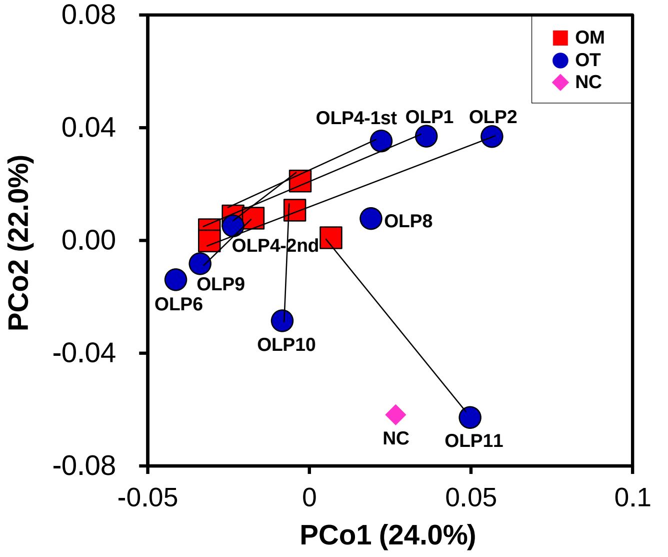

Supplementary Figure S1. A PCoA plot including the 2 tissue samples (OLP6 and OLP8) that lacked matching mucosal samples.

	OM							OT									
	OLP1	OLP2	OLP4-1st	OLP4-2nd	OLP9	OLP10	OLP11	OLP1	OLP2	OLP4-1st	OLP4-2nd	OLP9	OLP10	OLP11	OLP6	OLP8	NC
Streptococcus pneumoniae **group**	0.03	0	0.01	7.94	25.66	6.95	10.21	0	0	0	16.48	41.10	7.99	1.06	0.12	0.40	0.01
Haemophilus parainfluenzae **group**	19.99	9.31	6.75	16.95	25.39	12.66	1.81	8.47	5.27	3.03	12.49	8.00	3.99	0	0.32	27.93	0.01
Neisseria perflava	0.12	0.05	1.78	16.58	0.93	0.33	8.86	1.07	1.49	0.54	10.27	0.29	3.64	0.09	0	6.60	0
Lautropia mirabilis	1.11	2.25	2.86	1.37	8.27	0.83	8.96	3.37	0.22	0.11	0.03	1.76	0.05	0.35	0.53	0.04	0
Fusobacterium nucleatum **group**	1.02	0.59	1.34	2.47	1.99	9.55	7.38	0.54	0.15	0.11	0.01	1.39	0	0	0.55	0	0
Porphyromonas gingivalis	0.46	0.15	0.03	0	1.36	0.10	10.88	1.39	2.82	0.47	0	0	0	0	0.22	0	0
Streptococcus parasanguinis **group**	3.83	1.73	3.37	2.00	1.17	2.16	2.89	0.48	0.37	1.52	11.75	0.25	1.05	0	0.07	0	0
Rothia dentocariosa	0.28	0.18	7.52	12.89	1.93	3.98	0.32	0.20	0	0.93	0.24	0	0.85	0	0.24	0	0
Escherichia coli **group**	0.08	0.59	0.24	0	0	0.01	0	51.95	64.19	21.71	0	0	4.99	3.82	0.04	2.62	0.01
KV831974_s **group (*Rothia*)**	0	0	0	3.93	0.07	0.27	0.81	0	0	0	16.20	0	2.70	0	0	0	0.03
Bacteroides vulgatus	0	0	0	0	0	0.01	0	0	0	0	0	0	0	12.11	0	0	1.73
Streptococcus sanguinis **group**	1.04	8.80	5.60	0.71	1.18	5.28	2.89	0.91	0.67	0.45	0.07	0.78	0	0	0.62	0	0.01
Neisseria sicca **group**	0	1.43	0.33	0.07	0.22	3.04	3.62	0.04	0	0.02	0	1.44	0	0	0.15	0	0.32
Leptotrichia hongkongensis	0.04	0.07	1.64	1.11	0.31	10.17	0.26	0	0	1.01	0.66	2.34	0.50	0	0.51	0.08	0
Capnocytophaga leadbetteri	0.04	0.08	0.11	0.01	1.11	0.47	4.53	0.01	0	0	0	0.12	0	0	0.07	5.24	0
Rothia aeria	0.31	1.63	10.99	0.55	0.59	2.48	1.95	0.06	0.07	0.29	0.02	0	0	0	0	0	0
Streptococcus sinensis **group**	0	0	0	3.99	0.37	0.17	0.77	0	0	0	9.92	0.62	0.80	0	0	0	0.05
Streptococcus salivarius **group**	5.09	12.23	12.49	1.33	0.31	0.16	0.26	3.21	2.97	8.76	0.38	1.89	0	0	12.12	0.16	0
Gemella haemolysans **group**	1.67	1.46	0.24	0.48	6.88	0.80	0.94	0.04	0.15	0.06	0.15	8.94	0	0.38	4.20	2.62	0
Rothia mucilaginosa **group**	1.14	1.86	7.68	0	0.89	0.02	0	1.35	2.15	43.45	0.02	0	0	0	0.04	0.20	0
Streptococcus pseudopneumoniae	25.31	6.12	3.67	0	0	0	0	1.05	0.22	0.16	0	0	0	0	44.40	2.90	0
KE159600_s **(*Lachnospiraceae*)**	0	0	0	0	0	0	0	0	0	0	0	0	0	6.83*	0	0	0.40
JQ463704_s **(*Corynebacterium*)**	0	0	0	1.33	0.60	3.33	1.41	0	0	0	0	0	0	0	0	0	0
PAC001350_s **(*Leptotrichia*)**	0	0	0	1.43	0.19	5.76	0.17	0	0	0	0.25	0	0	0	0	0	0
Porphyromonas endodontalis	0	0.01	0.17	0	0.06	0.32	2.66	0.01	0	0	0	0	0	0	0.54	0	0
Lactobacillus plantarum **group**	0	0	0	0	0	0.02	0	0	0	0	0	0	4.99	0	0	0	15.29
Faecalibacterium prausnitzii **group**	0	0	0	0	0	0	0.01	0	0	0	0	0	0	4.84	0	0	1.45
Haemophilus parahaemolyticus **group**	0	0	0	0.01	1.08	2.50	1.09	0	0	0	0	3.98	0.15	0	0	0	0
Enterobacter_uc	0	0	0	0	0	0	0	0	0	0	0	0	0	4.97	0	0	0
Runella slithyformis	0	0	0	0	0	0	0	0	0	0	0	0	0	4.93	0	0	0
Aureimonas jatrophae **group**	0	0	0	0	0	0	0	0	0	0	0	0	0	4.86	0	0	0
Lachnospiraceae_uc_s	0	0	0	0	0	0	0	0	0	0	0	0	0	4.76*	0	0	0.06
Neisseria subflava	6.73	0.46	0.41	0.26	0.43	0.19	0.69	3.52	3.27	0.97	0.61	1.15	0	0	0.04	0	0
Mucispirillum schaedleri	0	0	0	0	0	0	0	0	0	0	0	0	0	4.19*	0	0	0.67
Megamonas rupellensis **group**	0	0	0	0	0	0	0	0	0	0	0	0	0	4.34*	0	0	0.17
AY134896_s **(*Leptotrichia*)**	0.01	0	0.01	0	0.10	4.94	0	0	0	0	0	0	0.10	0	0	0	0
Streptococcus dentisani	0.16	14.88	3.76	0	0	0	0	0.09	0.30	0.08	0	0	0	0	1.74	4.21	0
Weissella confusa **group**	0	0	0	0	0	0	0	0	0	0	0	0	0	3.86	0	0	0
Streptococcus anginosus **group**	0	0.18	0.29	0.19	0.56	0.02	0.04	0	0	0.05	0	1.27	0	2.80	0.23	0	0
Bacteroides coprophilus	0	0	0	0	0	0	0	0	0	0	0	0	0	3.15*	0	0	0.34
Microcystis aeruginosa **group**	0	0	0	0	0	0	0	0	0	0	0	0	0	3.02	0	0	0
Porphyromonas pasteri	0	0	0	0.36	0.52	0.06	0.27	0	0	0	3.31	0.12	0.20	0	0	0	0
PAC001149_s **(*Lachnospiraceae*)**	0	0	0	0	0	0	0	0	0	0	0	0	0	2.82	0	0	0
PAC001356_s **(*Leptotrichia*)**	0	0	0	0.39	0.12	0.03	0	0	0	0	4.07	5.33	0	0	0	0	0
PAC001244_s **(*Eubacterium_g8*)**	0	0	0	0	0	0	0	0	0	0	0	0	0	2.66	0	0	0
Streptococcus mitis	7.04	4.04	1.01	0	0	0	0	0.48	0.37	0.06	0	0	0	0	12.60	2.50	0
Lactobacillus helveticus **group**	0	0	0	0	0	0.01	0.002	0	0	0	0	0	0	0	0	0	6.63
PAC001304_s **(*Prevotella*)**	0	0	0	0	0	0	0	0	0	0	0	0	0	1.28*	0	0	2.64
Capnocytophaga_uc	0	0.03	0.02	0.04	0.30	0.10	0.09	0	0	0	2.59	0	0	0	0	0.16	0
CP006776_s **(*Streptococcus*)**	5.79	0.70	0.41	0	0	0	0	3.41	0.30	0.19	0	0	0	0	0	0	0
Neisseria oralis	0	5.30	1.27	0.19	0.45	0	0.01	0	0	0	0	0	0	0	0	0	0
Streptococcus infantis	3.44	0.93	1.11	0	0	0	0	3.48	0.82	0.42	0	0	0	0	0.09	2.42	0
Haemophilus paraphrohaemolyticus	1.44	3.15	0.12	0	0	0	0	0.01	0	0	0	0	0	0	7.73	0	0
Weissella kandleri **group**	0	0	0	0	0	0.23	0	0	0	0	0	0	27.06	0	0	0	0
AF432138_s **(*Leptotrichia*)**	0	0.06	0.24	0	0	0	0	0.04	0	3.15	0	0	0	0	0	0	0
Lactobacillus sakei **group**	0	0	0	0	0	0.02	0	0	0	0	0	0	7.94	0.01	0	0	0
Leuconostoc gelidum **group**	0	0	0	0	0	0.04	0	0	0	0	0	0	4.14	0	0	0	0.02
4P003122_s **(*Pasteurellaceae*)**	0.26	0.24	0.01	0	0	0	0	0	0	0	0	0	0	0	2.92	0	0

>50%	≥20%	≥10%	≥5%	≥2.5%	<2.5%

Supplementary Figure S2. A heat map showing dominant species that were present > 2.5% in any sample. An asterisk indicates contaminant.

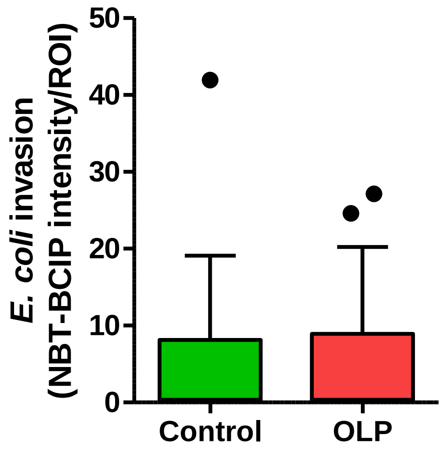

Supplementary Figure S3. Detection of *E. coli* invasion in control and OLP tissues. The mean intensity of bacterial signals in entire region including epithelia and lamina propria of control and OLP tissues was analyzed using ImageJ software. ROI, region of interest.

A

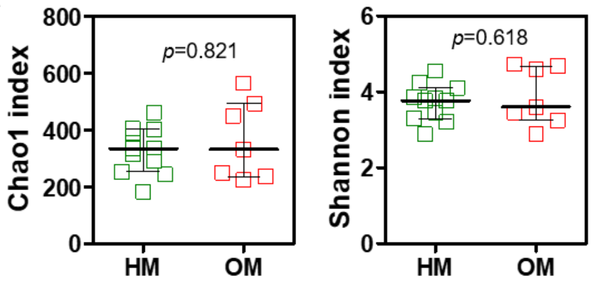

B

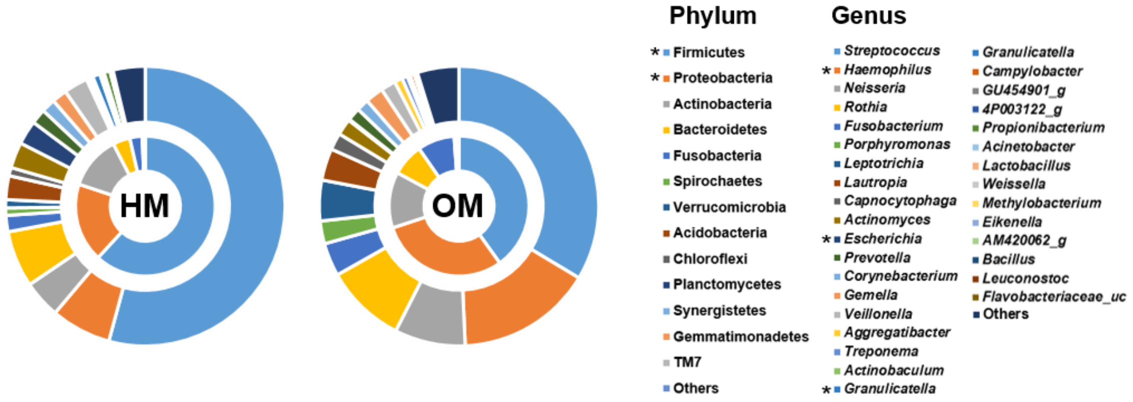

C

Species/phylotypes	HM (*n*=11)	OM (*n*=7)	*P*-value
Streptococcus pseudopneumoniae	18.73 (53.28)	0 (25.31)	0.012
Haemophilus parainfluenzae group	4.32 (15.26)	12.66 (23.58)	0.021
***Fusobacterium nucleatum* group**	0.19 (3.95)	1.99 (8.99)	0.026
***Escherichia coli* group**	0.45 (13.06)	0.01 (0.59)	0.007
HQ767899_s (Streptococcus)	2.40 (42.91)	0 (0)	0.001
KV831974_s group (*Rothia*)	0 (0)	0.07 (3.93)	0.006
Leptotrichia hongkongensis	0.02 (0.75)	0.31 (10.13)	0.009
Neisseria sicca group	0 (0.50)	0.33 (3.62)	0.010
Streptococcus sinensis group	0 (0)	0.17 (3.99)	0.006
Neisseria oralis	0 (0.03)	0.19 (5.30)	0.015
JQ463704_s (Corynebacterium)	0 (0)	0.60 (3.33)	0.006
PAC001350_s (Leptotrichia)	0 (0)	0.17 (5.76)	0.006
Aggregatibacter aphrophilus	0 (1.25)	0.47 (2.03)	0.010
Streptococcus dentisani	0 (0)	0 (14.88)	0.021
Haemophilus parahaemolyticus group	0 (0)	0.01 (2.50)	0.006
Fusobacterium periodonticum	0.70 (2.28)	0 (0.83)	0.032
Parvimonas micra	0.03 (0.20)	0.45 (1.20)	0.009
Granulicatella adiacens group	0.69 (2.67)	0.13 (0.75)	0.021
Treponema_uc	0 (0)	0.01 (1.29)	0.002
DQ016843_s (Streptococcus)	0.56 (3.05)	0 (0)	0.000
Porphyromonas pasteri	0 (0)	0.06 (0.52)	0.006
PAC001356_s (Leptotrichia)	0 (0)	0.06 (0.52)	0.006
Streptococcus rubneri	0 (0)	0 (2.15)	0.021
Aggregatibacter segnis	0 (0.23)	0.14 (0.68)	0.036
4P004975_s (Actinomyces)	0.04 (1.18)	0 (0)	0.002
Capnocytophaga sputigena	0.02 (0.06)	0.06 (0.72)	0.013
Streptococcus vestibularis	0.03 (0.26)	0 (0)	0.000
AF385572_s (Leptotrichia)	0 (0.04)	0.01 (0.08)	0.001
AY134896_s (Leptotrichia)	0 (0)	0.01 (4.94)	0.006
Streptococcaceae_uc_s	0.02 (0.05)	0 (0.02)	0.009
***Centipeda periodontii* group**	0 (0.03)	0.01 (0.04)	0.034
Selenomonas sputigena	0 (0.04)	0.02 (0.18)	0.020

Supplementary Figure S4. Altered mucosal microbiota in OLP. Bacterial communities of buccal mucosal samples of OLP lesions (n=7) were compared with those of healthy individuals (n=11) obtained in our previous study.[6] (A) The species richness and microbial diversity estimated by the Chao1 and Shannon diversity index, respectively (p by Mann-Whitney U test). (B) The double pie charts display the mean relative abundances of dominant phyla and genera (*$p < 0.05$ by Mann-Whitney U test). (C) Species/phylotypes differently distributed between the healthy and OLP groups (*$p < 0.05$ by Mann-Whitney U test) expressed as median and range. Species coincided with the previous results are bolded.

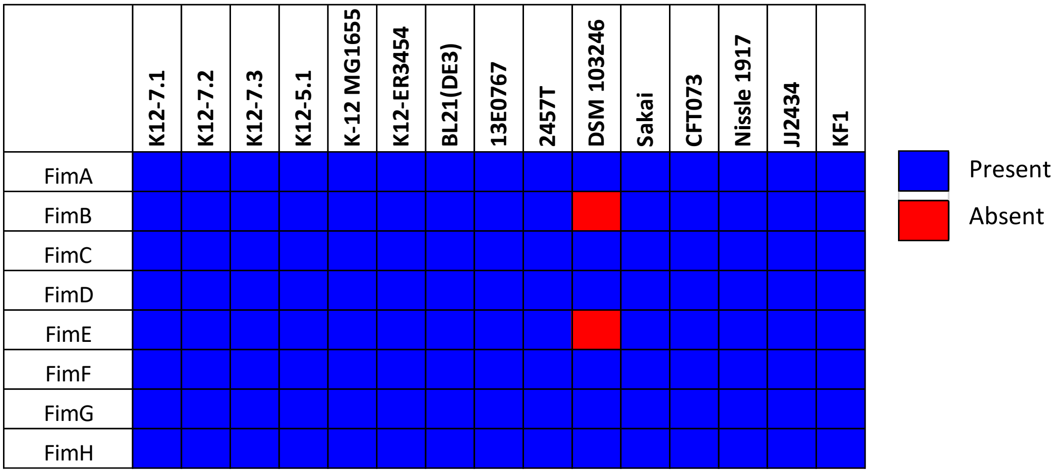

Supplementary Figure S5. Distribution of type 1 pilus-encoding genes

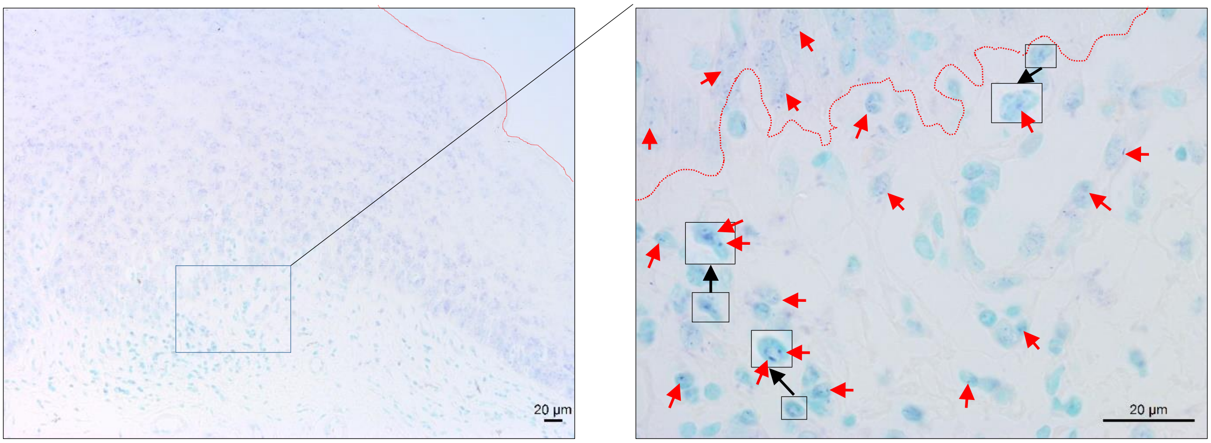

Supplementary Figure S6. Detection of *E. coli* by *in situ* hybridization in control tissue from a patient with suspected OLP Dotted red lines: outline of epithelium, red arrows: *in situ* signals for *E. coli*, rectangles: representative infected cells in the lamina propria are shown in higher magnification.
